# Primary Cardiac High-grade Myxofibrosarcoma Presenting with Multiple Brain Metastases: A Case Report

**DOI:** 10.7759/cureus.1866

**Published:** 2017-11-21

**Authors:** Filippo Badaloni, Eugenio Pozzati, Gianluca Marucci, Pietro Fiaschi, Antonio Fioravanti

**Affiliations:** 1 Department of Neurosurgery, IRCCS Institute of Neurological Sciences of Bologna, Bellaria Hospital, Bologna, Italy; 2 Neuropathology Unit, IRCCS Foundation “carlo Besta” Neurological Institute, Milan, Italy; 3 Department of Neurosciences, Rehabilitation, Ophthalmology, Genetics, Maternal and Child Health, University of Genoa, Genoa, Italy

**Keywords:** brain metastases, diagnosis, malignant cardiac tumors, unknown primary cancer, myxofibrosarcoma

## Abstract

Herein we describe the case of a young patient who presented with a recent history of epilepsy due to multiple brain lesions; he did not complain about any cardiopulmonary impairments. The patient died as a consequence of hemorrhagic progression of brain metastatic disease. Regardless of a thorough investigation, the heart tumor remained occult. Primary cardiac tumors are very rare entities. Most of these are benign, but approximately 25% are malignant, and the majority of these are sarcomas. Myxofibrosarcoma and osteosarcoma are exceptionally rare. To date, we find only small series of cardiac myxofibrosarcoma, and to our knowledge, this one exceptionally presented with multiple brain metastatic lesions without cardiopulmonary symptoms.

## Introduction

Primary cardiac tumors are very rare entities. The incidence is between 0.0017% and 0.03% in autopsy series. Most of the primary cardiac tumors are benign, but approximately 25% are malignant, and the majority of these are sarcomas. Angiosarcoma is the most frequent histotype, while myxofibrosarcoma and osteosarcoma are exceptionally rare. The prognosis of such malignancies is dismal. In case of complete or incomplete surgical resection of the primitive lesion, survival is 25 and 10 months, respectively [1]. Up to 80% of patients have evidence of metastases at the time of the initial presentation and the lung is the most common site [2-3]. These patients typically present with a history of dyspnea due to heart failure.

## Case presentation

**Figure 1:**
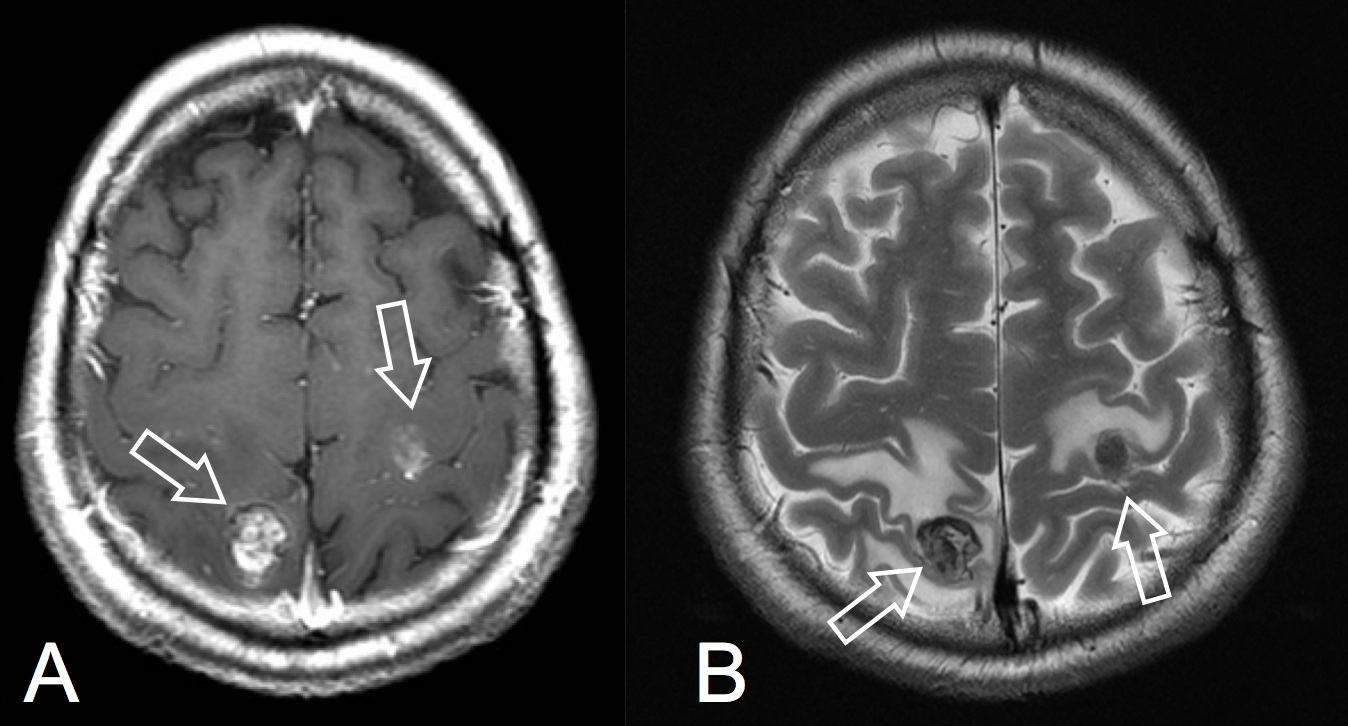
Preoperative MR axial images: A) post-gadolinium T1-W; B) T2-W. White arrows show the lesion. MR - magnetic resonance

A 41-year-old man was referred to our neurosurgical department after a one-month history of left side partial sensitive seizures and a recent onset of left side hemiparesis. His medical history was unremarkable. A post-contrast computed tomography (CT) scan showed multiple brain lesions: right side parietal (19 mm), left side rolandic (14 mm), and two subcentimetric lesions in the right temporal lobe. On magnetic resonance imaging (MRI), these lesions appeared as partially necrotic and hemorrhagic, with perilesional oedema and enhancement after gadolinium (Figures 1A-1B).

**Figure 2:**
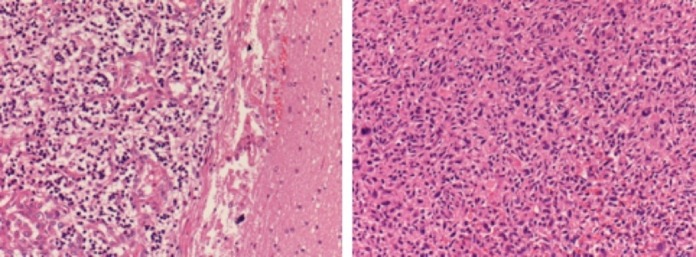
Histopathology: H&E 200X sections. Brain on the left and heart on the right.

The neoplastic serum markers were negative. A full-body CT scan did not evidence a primary tumor. A transthoracic echocardiography (TTE) and then a transesophageal echocardiography (TOE/TEE) were performed. A polilobulated (27X13 mm) mass was found, and it was attached to the mitral valve and partially occupying the left atrium. Low/moderate valve insufficiency was demonstrated. The lesion was echographically described as an incidental benign tumor such as a papillary fibroelastoma. Even an 18 fluorodeoxyglucose positron emission tomography–computed tomography (18-FDG PET-CT) scan did not show the primary tumor. As a consequence of the worsening of the neurological status (left side hemiparesis), regardless of the absence of the primitive tumor diagnosis, we decided to operate on the right parietal lesion. The patient underwent a neuronavigation-assisted right side parietal craniotomy. The lesion was resected “en-bloc” through a microsurgical trans-sulcal approach. A histological examination demonstrated a proliferation of atypical, spindle-shaped tumor cells with slightly eosinophilic cytoplasm and indistinct border, embedded in a myxoid matrix. The nuclei resulted hyperchromatic and pleomorphic, and atypical mitotic figures were observed (Figure 2). Neoplastic cells were immunopositive for vimentin and focally for smooth muscle actin. On the contrary, these cells were negative for melanocytic markers (S-100 protein, HMB45, MELAN-A), epithelial markers (CAM 5.2, EMA), vascular markers (CD31, CD34), as well as other muscular markers (myogenin, desmin). Thus a diagnosis of high-grade metastatic sarcoma was achieved.

**Figure 3:**
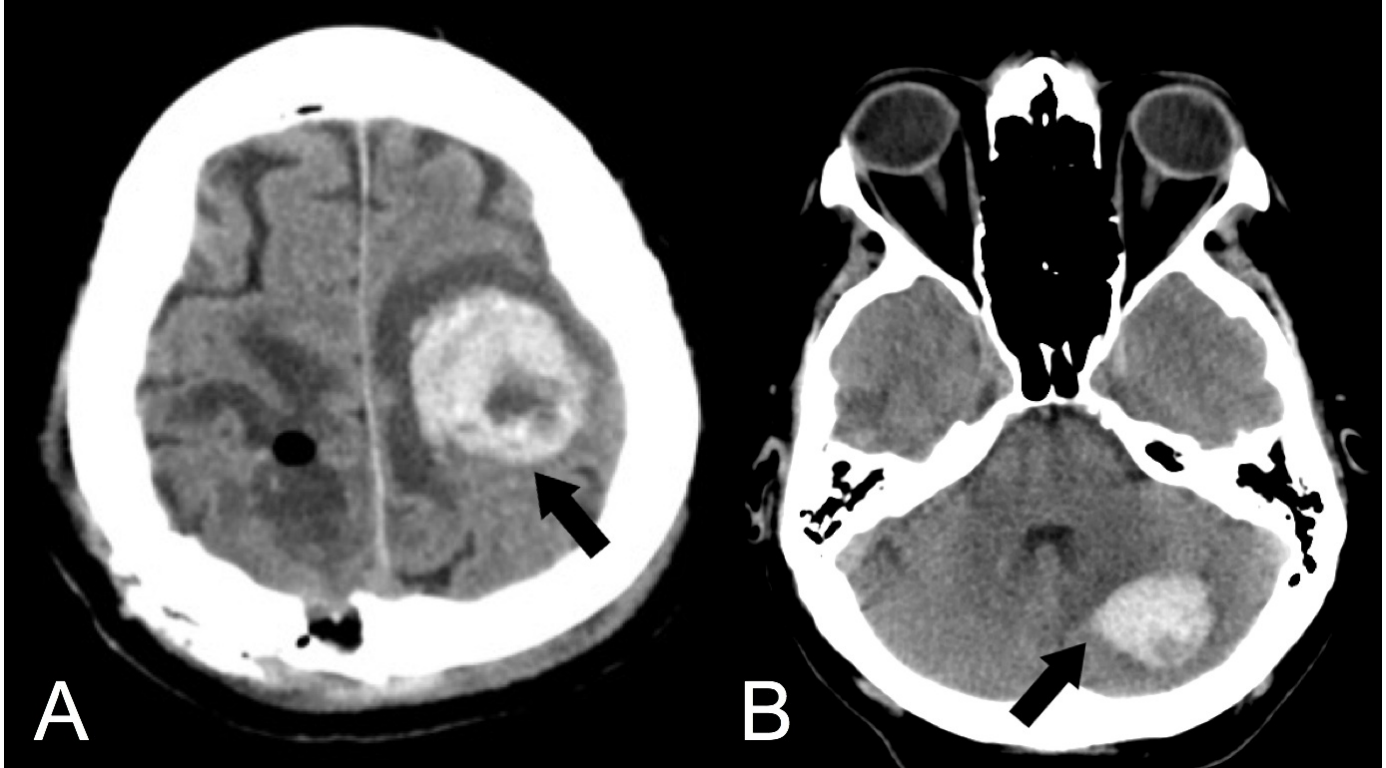
Late postoperative non-enhanced CT scan. A) Left side frontal hemorrhage; B) Left side cerebellar hemorrhage. Black arrows show spontaneous bleeding. CT - computed tomography

The early postoperative course was uneventful and the patient recovered left side hemiparesis, but the second day after surgery, a new onset of language impairment (motor aphasia) with right side hemiplegia suddenly appeared. A brain CT scan showed a haemorrhagic progression of the left rolandic lesion (Figure 3A). Later on, even a cerebellar lesion had haemorrhagic progression (Figure 3B).

**Figure 4:**
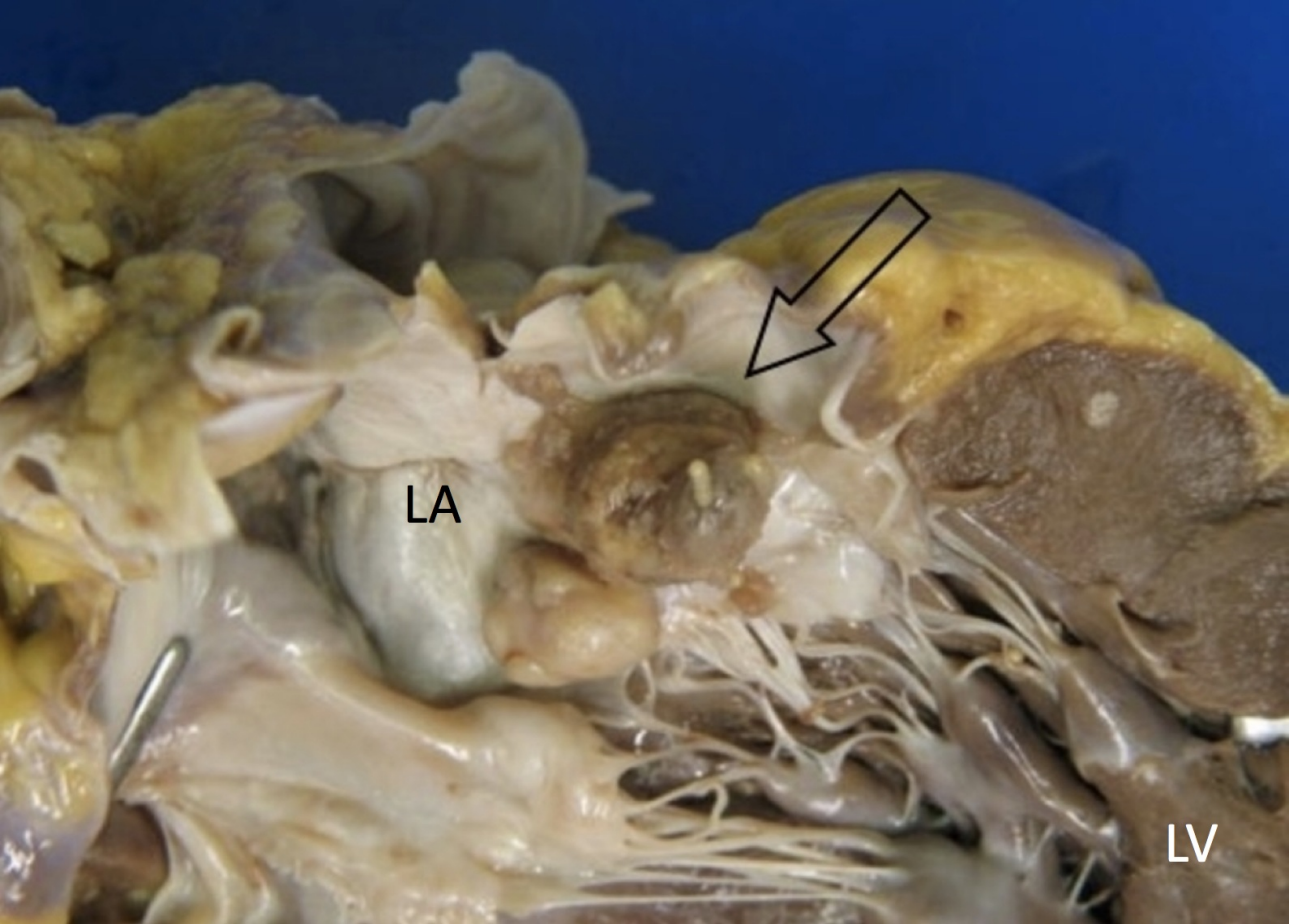
Heart - gross appearance. Arrow shows the tumor. LA - left atrium, LV - left ventricle

The patient passed away one month after surgery and seventy days after the onset of neurological symptoms. A postmortem examination revealed that the heart weighed 525 grams. We found two polypoid masses attached to the mitralic valve, measuring 15 mm and 10 mm, respectively, in maximum diameter, partially obstructing the left atrium (Figure 4). These lesions were composed of hard white tissue. Smaller nodules were found in the right and left ventricular wall. The brain dissection demonstrated multiple haemorrhagic lesions, located in the right parietal lobe (major axis 19 mm long), in the left rolandic area (30 mm), and in the right cerebellar lobe (40 mm). The final diagnosis was cardiac high-grade myxofibrosarcoma with multiple brain metastases.

## Discussion

Brain metastases are the most common brain tumor seen clinically. They develop in up to 35% of all cancer patients. It is well known that primary tumor histologic types, in the order of descending frequency, include non-small cell lung carcinoma (NSCLC), breast carcinoma, small cell lung carcinoma (SCLC), and malignant melanoma. On the other hand, up to 15% of newly diagnosed brain metastases patients have unknown primary tumor despite a thorough investigation [4]. Primary malignant cardiac tumors are extremely rare; they typically present with advanced tumor stage and nearly 80% of patients had metastases at the time of primary diagnosis [2]. They are clinically symptomatic with four main mechanisms: obstruction to blood flow and valvular dysfunction, local invasion causing arrhythmia or pericardial effusion, embolism, or systemic symptoms of dyspnea, fever, malaise, and weight loss [2]. Angiosarcoma is the most common histotype and lung is the most frequent site of metastases. They affect predominantly the right heart. The prognosis of such malignancies is dismal. In case of incomplete or complete resection of the cardiac tumor, survival may vary from 10 to 25 months, respectively [2, 5-6]. Unclassified sarcoma is another common name for myxofibrosarcoma, and such sarcomas affect predominantly the left atrium [5]. Brain metastasis from cardiac tumors represent an extremely rare event as well.

Myxofibrosarcoma is a mesenchymal neoplasia occurring most commonly in the extremities. More infrequently, this tumor may arise in other sites [5]. Few cases of cardiac myxofibrosarcomas have been reported in the literature [7-9]. Even if low-grade myxofibrosarcomas could recur locally, it is known that high-grade cases are associated with distant metastases, including brain metastases [3, 6, 10]. Nevertheless, TOE and thoracic CT/MRI scans are the gold standard to study cardiac masses, as it is extremely difficult to identify myxofibrosarcomas preoperatively [7], especially in those very atypical patients who do not express signs and symptoms of heart disease. Furthermore, 18 FDG PET/CT may not demonstrate the primary tumor, due to the high metabolic activity of heart tissue. In this case, a history of cardiopulmonary disease was exceptionally absent despite the fact that primary cardiac malignancy was constituted by large multiple neoplastic masses. On the contrary, the patient presented with focal neurological impairment secondary to cerebral mass effect. Furthermore, this case demonstrates that cerebral metastases from myxofibrosarcoma have a high tendency to evoke a haemorrhagic course with ominous effects on overall survival.

## Conclusions

In conclusion, albeit their occurence is extremely rare, the recognition of brain metastases from myxofibrosarcomas and their distinction from other hemorrhagic tumors may have therapeutic and prognostic implications.

## References

[1] Devbhandari MP, Meraj S, Jones MT (2007). Primary cardiac sarcomas: reports of two cases and a review of current literature. J Cardiothorac Surg.

[2] Awamleh P, Alberca MT, Gamallo C (2007). Left atrium myxosarcoma: an exceptional cardiac malignant primary tumor. Clin Cardiol.

[3] Kim DG, Lee SY, Chung SK (1997). Brain metastasis from myxofibrosarcoma of the heart. Acta Neurochir.

[4] Alexandrua D, Bota DA, Linskey ME (2012). Epidemiology of central nervous system metastases. Prog Neurol Surg.

[5] Kim CH, Dancer JY, Coffey D (2008). Clinicopathologic study of 24 patients with primary cardiac sarcomas: a 10-year single institution experience. Hum Pathol.

[6] Jung SH, Jung TY, Joo SP, Kim HS (2012). Rapid clinical course of cerebral metastatic angiosarcoma from the heart. J Korean Neurosurg Soc.

[7] Heletz I, Abramson SV (2009). Large obstructive cardiac myxofibrosarcoma is nearly invisible on transthoracic echocardiogram. Echocardiography.

[8] Huang HY, Lal P, Qin J (2004). Low grade myxofibrosarcoma: a clinicopathological analysis of 49 cases treated at a single institution with simultaneous assessment of the efficacy of 3-tier and 4-tier grading systems. Hum Pathol.

[9] Lazaros GA, Matsakas EP, Madas JS (2008). Primary myxofibrosarcoma of the left atrium: case report and review of the literature. Angiology.

[10] Regel JP, Pospiech J, Baume B (2006). Cerebral metastasis from an undifferentiated sarcoma of the left atrium. Acta Neurochir.

